# ICU-acquired weakness, diaphragm dysfunction and long-term outcomes of critically ill patients

**DOI:** 10.1186/s13613-019-0618-4

**Published:** 2020-01-03

**Authors:** Clément Saccheri, Elise Morawiec, Julie Delemazure, Julien Mayaux, Bruno-Pierre Dubé, Thomas Similowski, Alexandre Demoule, Martin Dres

**Affiliations:** 1 Service de Pneumologie, Médecine intensive – Réanimation (Département “R3S”), AP–HP, Sorbonne Université, Groupe Hospitalier Universitaire Pitié-Salpêtrière Charles Foix, 75013 Paris, France; 2 Département de médecine, service de pneumologie, hôpital Hôtel-Dieu du Centre Hospitalier de l’Université de Montréal, Montreal, QC Canada; 3 Centre de Recherche du Centre Hospitalier de l’Université de Montréal – Carrefour de l’Innovation et de l’Évaluation en santé, Montreal, QC Canada; 4 UMRS1158 Neurophysiologie respiratoire expérimentale et clinique, Sorbonne Université, Paris, France

**Keywords:** Diaphragm dysfunction, Survival, Limb muscle weakness, Mortality, Quality of life

## Abstract

**Background:**

Intensive care unit (ICU)-acquired weakness and diaphragm dysfunction are frequent conditions, both associated with poor prognosis in critically ill patients. While it is well established that ICU-acquired weakness severely impairs long-term prognosis, the association of diaphragm dysfunction with this outcome has never been reported. This study investigated whether diaphragm dysfunction is associated with negative long-term outcomes and whether the coexistence of diaphragm dysfunction and ICU-acquired weakness has a particular association with 2-year survival and health-related quality of life (HRQOL).

**Methods:**

This study is an ancillary study derived from an observational cohort study. Patients under mechanical ventilation were enrolled at the time of their first spontaneous breathing trial. Diaphragm dysfunction was defined by tracheal pressure generated by phrenic nerve stimulation < 11 cmH_2_O and ICU-acquired weakness was defined by Medical Research Council (MRC) score < 48. HRQOL was evaluated with the SF-36 questionnaire.

**Results:**

Sixty-nine of the 76 patients enrolled in the original study were included in the survival analysis and 40 were interviewed. Overall 2-year survival was 67% (46/69): 64% (29/45) in patients with diaphragm dysfunction, 71% (17/24) in patients without diaphragm dysfunction, 46% (11/24) in patients with ICU-acquired weakness and 76% (34/45) in patients without ICU-acquired weakness. Patients with concomitant diaphragm dysfunction and ICU-acquired weakness had a poorer outcome with a 2-year survival rate of 36% (5/14) compared to patients without diaphragm function and ICU-acquired weakness [79% (11/14) (*p* < 0.01)]. Health-related quality of life was not influenced by the presence of ICU-acquired weakness, diaphragm dysfunction or their coexistence.

**Conclusions:**

ICU-acquired weakness but not diaphragm dysfunction was associated with a poor 2-year survival of critically ill patients.

## Background

Critically ill patients are likely to develop respiratory and limb muscle weakness [1]. It has been clearly established that intensive care unit (ICU)-acquired limb muscle weakness is a frequent condition, associated with poor prognosis [2–6]. Although the consequences of respiratory muscle weakness have been less extensively investigated, some evidences suggest that it may be a risk factor for prolonged duration of mechanical ventilation [7], associated with a higher risk of readmission [8] and higher mortality [9]. Diaphragm dysfunction is frequent in the intensive care unit (more than 60% of mechanically ventilated patients [10]) and is associated with difficult and prolonged weaning and poor prognosis [11–14]. Causes of diaphragm dysfunction are multifactorial and no specific treatment is currently available [10]. Diaphragm dysfunction is responsible for prolonged mechanical ventilation and may contribute to the development of “chronic critical illness”, a devastating condition for patients and their families [15]. While the diaphragm undoubtedly plays a crucial role to ensure prompt and safe weaning from mechanical ventilation, recent data suggest that a substantial proportion of patients (up to 44%) can be successfully weaned from the ventilator despite the presence of diaphragm dysfunction [13, 14, 16]. Nevertheless, in contrast with the reported negative impact of ICU-acquired weakness on long-term survival and disabilities [3, 4, 17], it remains unclear whether diaphragm dysfunction per se is responsible for poor medium-term and long-term prognosis. The present study therefore used a previous cohort study conducted in our institution [13] to follow up mechanically ventilated patients in whom diaphragm and limb muscle functions were investigated at the time of liberation from mechanical ventilation. We tested the hypothesis that diaphragm dysfunction is associated with negative long-term outcomes and that the coexistence of diaphragm dysfunction and ICU-AW has a particular association on long-term prognosis, in line with the known negative synergistic effects of the two conditions on short-term prognosis.

## Methods

This study was conducted in a 10-bed medical ICU in a university hospital. ICU patients were enrolled over an 8-month period and phone interviews were conducted over a 4-month period. The study was approved by the Comité de Protection des Personnes Ile-de-France VI (ID RCB: 2014-A00715-42) and was conducted in accordance with the ethical standards laid down in the 2008 Declaration of Helsinki. Informed consent was obtained from all patients or their relatives. Some data from this cohort have already been published elsewhere [13, 18, 19].

### Initial study population

As described elsewhere [13], patients were eligible for the study if they had been intubated and ventilated for at least 24 h and met the readiness-to-wean criteria as routinely assessed by the clinicians in charge on a daily basis according to current guidelines [20]. Patients presenting factors possibly interfering with tracheal pressure measurements in response to phrenic nerve stimulation (chest tubes, neuromuscular disease), tracheostomy, a contraindication to the phrenic nerve stimulation technique (pacemaker) and patients with withholding or withdrawal of life support decisions at the time of inclusion were not enrolled.

Diaphragm function and limb muscle strength were assessed on the same day once after enrolment and before starting the first spontaneous breathing trial. Diaphragm function was assessed by the change in tracheal pressure in response to magnetic stimulation (Ptr,stim), as described previously [13] and limb muscle strength was assessed by the Medical Research Council (MRC) score in patients screened for level of consciousness and understanding [2, 21]. Patients with a Ptr,stim less than 11 cm H_2_O were considered to have diaphragm dysfunction and patients with a Ptr,stim less than 7 cmH_2_O were considered to have severe diaphragm dysfunction [18]. Patients with an MRC score less than 48 were considered to have ICU-AW [2].

### Data collection for long-term prognosis study

Variables pertaining to patient characteristics and ICU stay were collected prospectively during hospitalization: age, sex, comorbidities, the Simplified Acute Physiology Score (SAPS II) and SOFA score computed at ICU admission [22, 23]. The dates of ICU admission, intubation, first spontaneous breathing trial, extubation, ICU discharge, hospital discharge and ICU and hospital death were obtained from the hospital medical charts. Survival and health-related quality of life assessment was performed as described below. Hospital medical charts were analysed to identify the patient’s status (dead or alive) and dates over a 2-year period following inclusion in the study. Patients alive 2 years after inclusion were contacted by phone by the first author. The patient was asked to complete the Medical Outcomes Study 36-Item Short-Form Health Survey (SF-36) questionnaire to assess health-related quality of life [24, 25]. The SF-36 questionnaire is a general quality of life questionnaire that evaluates physical and emotional quality of life. The SF-36 questionnaire includes eight multiple-item scales that assess physical functioning, social functioning, physical role, emotional role, mental health, pain, vitality, and general health [26]. Interviews were conducted by a single investigator blinded to the patient’s status in relation to the presence of diaphragm dysfunction and ICU-acquired weakness in the ICU.

### Study outcomes

The primary outcome was 2-year overall survival following inclusion, and secondary outcomes were health-related quality of life and functional disability as assessed by the SF-36 score.

### Statistical analysis

Due to the retrospective nature of this post hoc analysis, no sample size calculation could be made. All patients from the primary cohort [13] with available data were included in the present analysis. Continuous variables are expressed as median (interquartile range) and categorical variables are expressed as absolute and relative frequency. A normal distribution was checked by a Kolmogorov–Smirnov test. Continuous variables were compared by Mann–Whitney test and categorical variables were compared by Fisher’s exact test. Kaplan–Meier survival curves were generated to describe cumulative survival following inclusion. Survival curves were compared by log-rank (Mantel–Cox) test.

Patient outcomes were compared by means of two classifications. First, patients were classified as follows: (1) with/without diaphragm dysfunction and (2) with/without ICU-acquired weakness. In a secondary analysis, patients were also classified according to the co-existence of diaphragm dysfunction and ICU-acquired weakness: (a) diaphragm dysfunction only, (b) ICU-acquired weakness only, (c) co-existence of diaphragm dysfunction and ICU-acquired weakness and (d) neither of these two conditions.

Three exploratory analyses were then conducted. A first exploratory analysis assessed the 2-year survival in hospital survivors and a second exploratory analysis assessed the 2-year survival in patients with or without severe diaphragm dysfunction (Ptr,stim < 7 cmH_2_O). Last, a third exploratory analysis compared SF-36 questionnaire in patients with co-existence of diaphragm dysfunction and ICU-acquired weakness and those who were free from diseases. For all final comparisons, a *p*-value less than or equal to 0.05 was considered statistically significant. Statistical analyses were performed with Prism v.6, GraphPad Software.

## Results

### Patient characteristics and follow-up

**Fig. 1:**
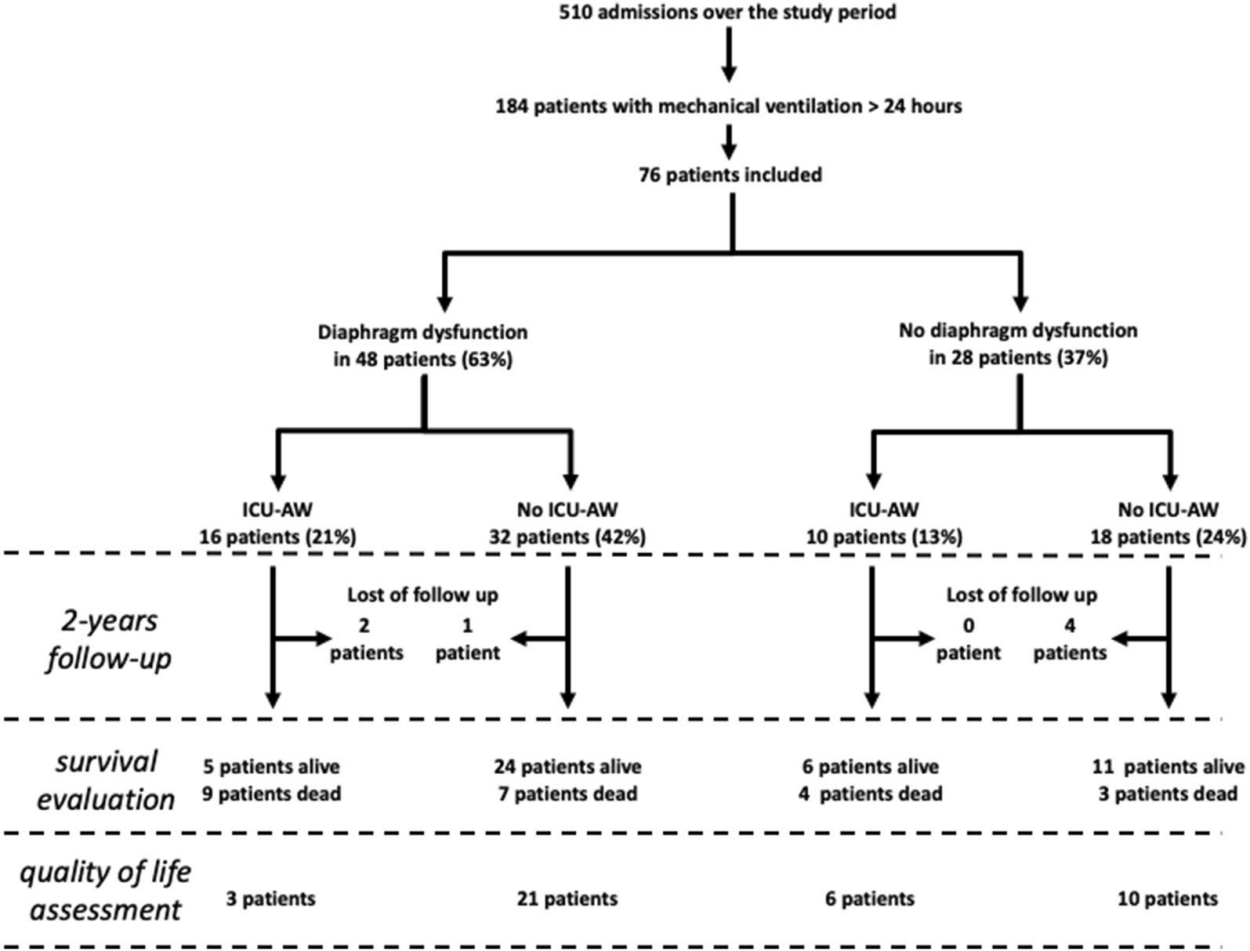
Study flowchart. *ICU-AW* intensive care unit-acquired weakness

Seventy-six patients were consecutively enrolled in the primary study (Fig. 1). Forty-eight (63%) patients were diagnosed with diaphragm dysfunction at the time of weaning, 26 (34%) had ICU-acquired weakness, 16 (21%) had both diaphragm dysfunction and ICU-acquired weakness and 18 (24%) were free of diseases. Patient characteristics are presented in Table 1. Patients had been ventilated for an average of 4 (2–6) days prior to inclusion. Patients who were free from diaphragm dysfunction and ICU-AW were more likely placed under mechanical ventilation for coma and were ventilated significantly shorter than others (Table 1). Overall, 43 (57%) patients were successfully weaned from the ventilator after the first attempt. The mean length of ICU stay was 9 (4–18) days and the mean length of hospital stay was 21 (10–30) days. All ICU survivors had been liberated from mechanical ventilation at the time of ICU discharge. As 7 patients were lost to follow-up, 2-year survival was assessed in only 69 patients and secondary outcomes (health-related quality of life) were assessed in 40 patients (Fig. 1).

**Table 1:** Characteristics and main outcomes of the study’s population according to the presence of diaphragm dysfunction and intensive care unit-acquired weakness (ICU-AW) at time of liberation from mechanical ventilation

Characteristics and outcomes	Diaphragm dysfunction		No diaphragm dysfunction		*p*
	No ICU-AW *N* = 32	ICU-AW *N* = 16	No ICU-AW *N* = 18	ICU-AW *N* = 10	
Men, *n* (%)	20 (63)	12 (75)	15 (83)	5 (50)	0.23
Age, years	63 (47–74)	65 (54–72)	47 (33–64)	60 (51–68)	0.05
SAPS II upon admission	44 (24–64)	43 (28–67)	32 (15–42)	37 (26–63)	0.18
SOFA upon admission	4 (3–7)	5 (5–7)	5 (4–5)	6 (4–10)	0.47
Duration of MV at inclusion, days	4 (1–6)	6 (4–10)^a^	2 (1–5)	5 (3–6)	<0.01
Main reason for admission, *n* (%)					
Acute respiratory failure	17 (53)	8 (50)	3 (17)	3 (30)	0.06
Coma	2 (6)	4 (25)	13 (72)	3 (30)	<0.01
Shock	13 (41)	4 (25)	2 (11)	4 (40)	0.14
Comorbidities, *n* (%)					
Chronic respiratory disease	8 (25)	2 (13)	2 (11)	0 (0)	0.23
Chronic cardiac disease	7 (22)	3 (19)	2 (11)	0 (0)	0.36
Diabetes	5 (16)	4 (25)	4 (22)	1 (10)	0.74
Immunocompromised	6 (19)	6 (38)	3 (16)	3 (30)	0.42
Current smoking	18 (56)	7 (44)	7 (39)	5 (50)	0.66
Limbs muscles strength					
Medical Research Council score	54 (51–57)	36 (30–44)	59 (52–60)	40 (33–44)	–
Diaphragm function					
Ptr,stim, cmH_2_O	7 (6–8)	6 (3–8)	14 (13–20)	12 (11–17)	–
Main outcomes					
Total duration of MV, days	6 (3–12)	10 (5–22)^a^	2 [1–5]	7 (5–20)^a^	< 0.01
Duration of ICU stay, days	9 (3–16)	17 (7–27)^a^	4 [3–10]	13 (8–27)	< 0.01
Duration of hospital stay, days	21 (15–31)	25 (15–49)^a^	9 [5–23]	27 (13–32)	0.03
ICU survival, *n* (%)	27/32 (84)	10/16 (69)	18/18 (100)	8/10 (80)	0.33
Hospital survival, *n* (%)	26/32 (81)	9/16 (63)	18/18 (100)	8/10 (80)	0.02
Two-year survival, *n* (%)	24/31 (77)	5/14 (36)	11/14 (79)	6/10 (60)	0.03

Categorical variables are expressed as absolute value (%) and continuous variables are expressed as median (interquartile range [25–75%])

*p* values according to Kruskal–Wallis test for continuous variables and Chi-2 for dichotomous variables

*SAPS 2* Simplified Acute Physiology Score, *SOFA* Sequential Organ Failure Assessment score, *MV* mechanical ventilation, *ICU* intensive care unit, *ICU-AW* ICU-acquired weakness, *Ptr,stim* tracheal pressure during the phrenic nerves stimulation

^a^As compared to the group of 18 patients without diaphragm dysfunction neither ICU-AW

### Two-year survival

**Fig. 2:**
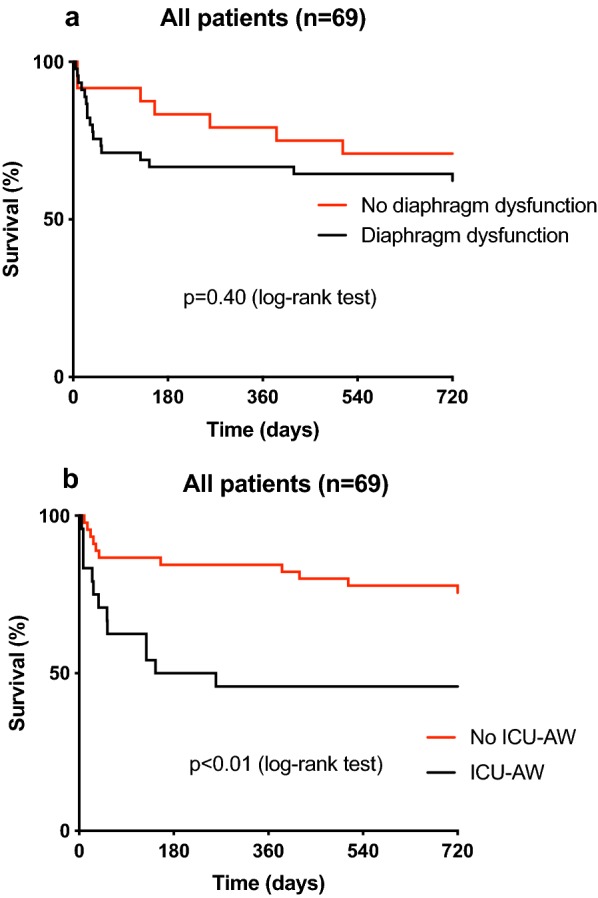
Kaplan–Meier 2-year survival curves in patients with and without diaphragm dysfunction (**a**) and in patients with and without ICU-AW (**b**)

The overall 2-year survival was 67% (46/69): 64% (29/45) in patients with diaphragm dysfunction, 71% (17/24) in patients without diaphragm dysfunction, 46% (11/24) in patients with ICU-acquired weakness and 76% (34/45) in patients without ICU-acquired weakness. Patients with co-existence of diaphragm dysfunction and ICU-acquired weakness had poorer survival than patients without diaphragm function and ICU-acquired weakness (*p* = 0.01 for overall comparison, see Additional file 1: Figure S1). Survival was not significantly different between patients with diaphragm dysfunction and patients without diaphragm dysfunction (see Fig. 2a), but was significantly poorer in patients with ICU-acquired weakness compared to patients without ICU-acquired weakness (see Fig. 2b).

#### Exploratory analysis restricted to hospital survivors

**Fig. 3:**
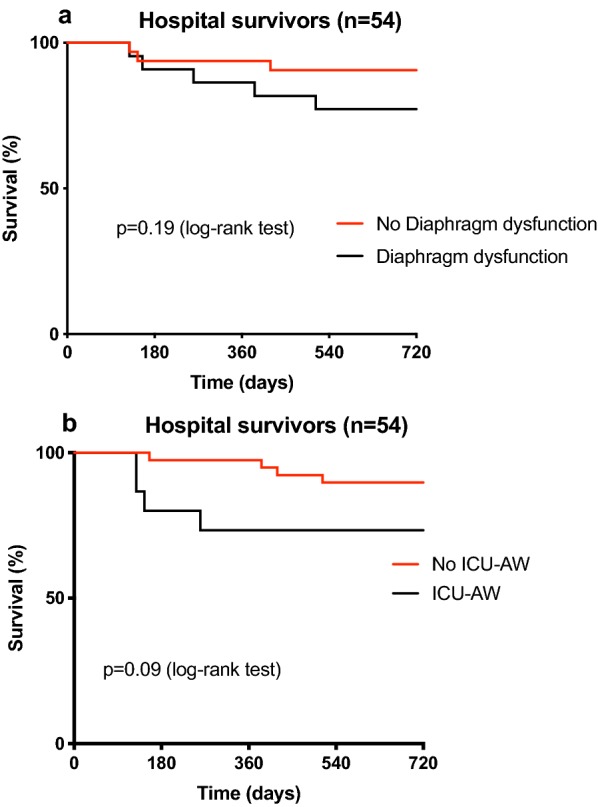
Kaplan–Meier 2-year survival curves in patients with and without diaphragm dysfunction (**a**) and in patients with and without ICU-AW (**b**) (sensitivity analysis restricted to hospital survivors)

Among the 76 patients enrolled in the study, 61 were alive at the time of hospital discharge. From them, 7 were lost of follow-up leading to a sample of 54 patients in the exploratory analysis (see Fig. 3 and Additional file 1: Figure S2). Although the comparison between groups was not significant, the findings were in line with the primary analysis.

#### Exploratory analysis conducted on patients with severe diaphragm dysfunction

A severe diaphragm dysfunction (Ptr,stim < 7 cmH_2_O) was diagnosed in 31% (24/76) of the patients. The survival between patients with and without severe diaphragm dysfunction was not significantly different (*p* = 0.07) (see Additional file 1: Figure S3).

### Health-related quality of life

**Fig. 4:**
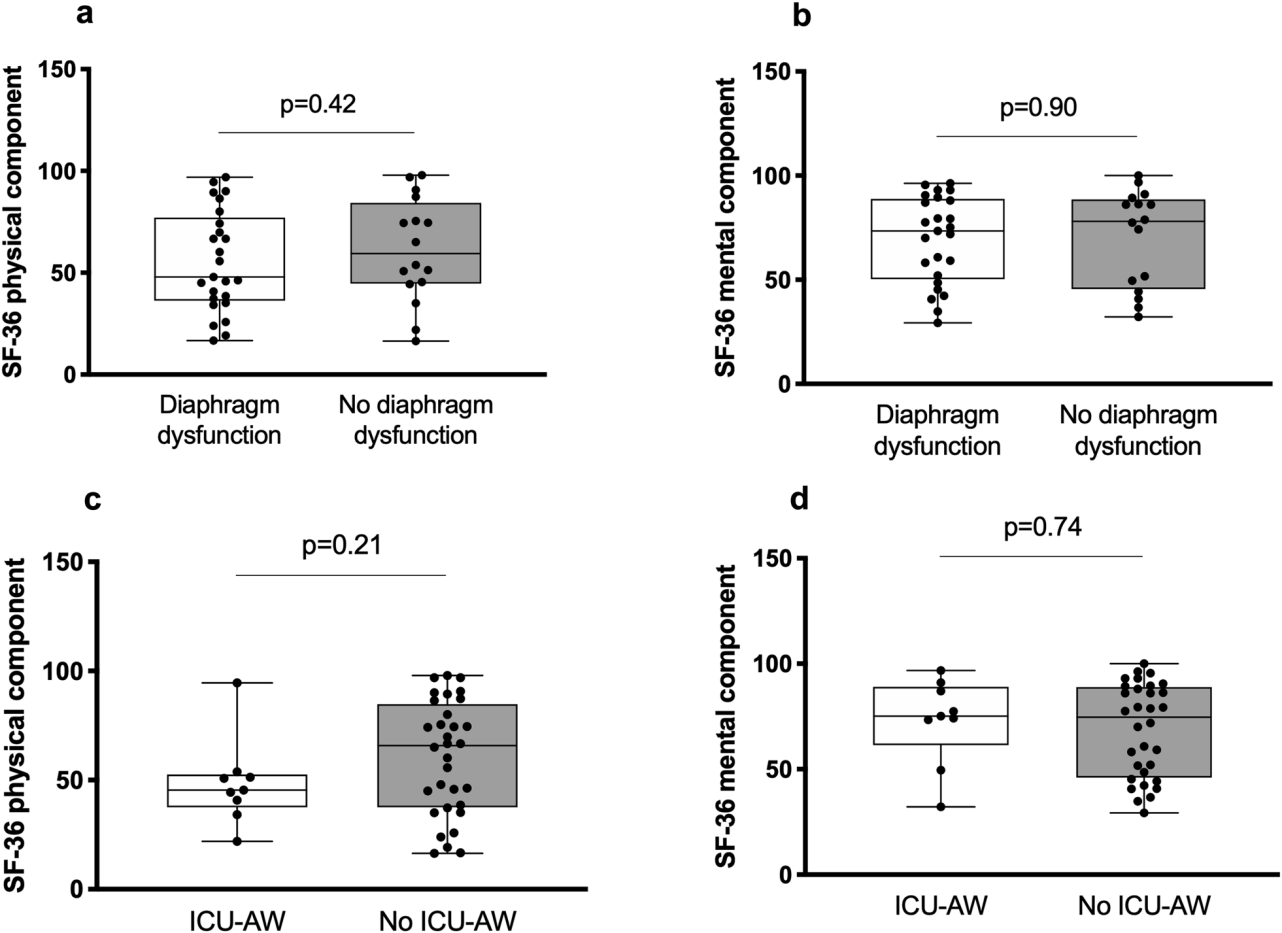
SF-36 physical component scores at 2 years among patients with and without diaphragm dysfunction (**a**) and patients with and without intensive care unit-acquired weakness (ICU-AW) (**c**) and SF-36 mental component scores among patients with and without diaphragm dysfunction (**b**) and patients with and without ICU-AW (**d**) (*n* = 40 patients)

The SF-36 questionnaire was completed at the end of the study in 40 patients. Figure 4 displays the values of the SF-36 components (mental and physical) in patients with and without diaphragm dysfunction and in patients with and without ICU-acquired weakness. No significant difference in terms of SF-36 categories was observed between groups. The exploratory analysis comparing patients with co-existence of diaphragm dysfunction and ICU-acquired weakness and those who were free from diseases found no difference in term of SF-36 mental and physical components (see Additional file 1: Figure S4).

## Discussion

This study reports the long-term—up to 2 years—survival and health-related quality of life of critically ill patients with diaphragm dysfunction, ICU-acquired weakness or a combination of the two at the time of liberation from mechanical ventilation. The main findings can be summarized as follows: (1) 2-year survival was much better in patients without diaphragm dysfunction and ICU-acquired weakness at the time of liberation from mechanical ventilation than in patients presenting both of these conditions (79% versus 36%, respectively); (2) survival was not significantly different between patients with diaphragm dysfunction without ICU-acquired weakness and patients not presenting either of the two conditions (77% versus 79%, respectively); (3) survival appeared to be much more markedly influenced by the presence of ICU-acquired weakness than by the presence of diaphragm dysfunction and (4) the study failed to demonstrate any difference in terms of health-related quality of life according to the presence of diaphragm dysfunction and/or ICU-acquired weakness.

Only a few studies have examined the association between ICU-acquired weakness and long-term survival. Sharshar et al. reported an in-hospital survival of 77% in patients with ICU-acquired weakness [17] and Ali et al. reported an in-hospital survival of 69% [3]. In a study with prolonged follow-up (up to 1 year), Hermans et al. reported a 68% survival rate in patients with ICU-acquired weakness [4]. In our study, patients with ICU-acquired weakness had a 2-year survival of 46%. This lower survival rate in our cohort could be explained by the timing of the assessment of ICU-acquired weakness (earlier in our study than in other studies) and the prolonged duration of follow-up. It remains unclear whether diaphragm dysfunction is one of several features of ICU-acquired weakness or whether the two conditions are distinct [7, 13, 27]. Two studies conducted at the time of weaning demonstrated a weak but significant correlation between diaphragm function and limb muscle strength [13, 14]. One of these studies also suggested a difference in prognosis between the two conditions, as ICU-acquired weakness was more strongly associated with prolonged hospital stay and duration of mechanical ventilation, while diaphragm dysfunction was mostly associated with difficult/prolonged weaning and ICU and hospital mortality [13]. The present study reports novel findings in this context, suggesting that ICU-acquired weakness may be associated with a more severe impact on long-term outcome than diaphragm dysfunction. The association of diaphragm dysfunction with long-term prognosis has never been previously investigated. One study investigated the impact of global respiratory weakness (as assessed by the maximal inspiratory pressure) and reported a higher 1-year mortality (31%) in the group of patients with low maximal inspiratory pressure compared to patients with high maximal inspiratory pressure, in whom the 1-year mortality was 7% [9]. However, limb muscle strength was not analysed in this study. Our findings suggest that the long-term prognosis of these patients is mainly driven by the presence of ICU-acquired weakness rather than diaphragm dysfunction. Nevertheless, as expected, the co-existence of these two conditions was associated with a poor survival. The presence of diaphragm dysfunction in critically ill patients has been associated with poor short-term prognosis in some studies [11, 13, 14, 28], but not in others [29, 30]. Remarkably, a recent study demonstrated that diaphragm dysfunction was not associated with an increased risk of extubation failure in patients who successfully passed the weaning trial [29], which is in line with our findings, suggesting that diaphragm function is a critical determinant in the outcome of the weaning trial, but once patients have been weaned from the ventilator, the long-term prognosis is mainly determined by other risk factors, notably ICU-acquired weakness.

Our study also investigated the respective association of ICU-acquired weakness and diaphragm dysfunction with health-related quality of life and indicated that health-related quality of life was not significantly different 2 years after the ICU stay regardless of the presence or absence of diaphragm dysfunction or ICU-acquired weakness. These findings are in contrast with previous data reporting substantial impairments in physical function and health-related quality of life in acute lung injury survivors [31]. This discrepancy may be explained by the timing of ICU-acquired weakness evaluation (after a few days of mechanical ventilation in our study as compared to 3 months after the acute lung injury in the study by Fan et al.) and the different period of time for both investigations (2014–2015 in our study versus 2004–2007 in the study by Fan et al.) [31]. Although not statistically significant, the SF-36 questionnaire score indicated better health-related quality of life in patients not presenting either of these two conditions. Importantly, this difference may be underestimated by the competitive risk of death. The findings of this study could possibly be explained by a lack of power, but they could also be the consequence of self-adaptive quality of life perception [32]. Eventually, the findings on SF-36 questionnaire should be considered as an exploratory analysis according to the sample size and warrant further studies.

### Strengths and limitations

The main strength of our study is the phrenic nerve stimulation that was used to evaluate diaphragm function, and this method is considered to be the reference method in the ICU. In addition, our study provides insight into the association of diaphragm dysfunction and ICU-acquired weakness with survival and quality of life over a long period of time after the ICU stay. However, some limitations must be acknowledged. The first and main limitation concerns the small sample size finally analysed. Indeed, this is a cohort study with a significant risk of confounding and relatively small event rates. As such the statistical analyses do not adjust for potential confounding and explore simple associations. Our findings must therefore be interpreted cautiously and larger studies are warranted. Secondly, diaphragm function and limb muscle strength were not repeatedly assessed in our study. Consequently, the findings in terms of survival and quality of life could not be correlated with possible recovery of either diaphragm function or limb muscle strength. Thirdly, ICU-acquired weakness was assessed with MRC score that relies on the cooperation of the patients. Therefore, even if patients were confirmed awake, it is a volitional measurement that may be associated with a ceiling effect and poor responsiveness. Fourthly, due to small size of the cohort, competing risk of death could not be take into account which may have created a bias in the assessment of health care quality of life.

## Conclusion

At the time of starting the process of weaning from mechanical ventilation, diaphragm dysfunction and ICU-acquired weakness were associated with distinct outcomes. ICU-acquired weakness was associated with a poorer 2-year survival of critically ill patients than diaphragm dysfunction, whereas the presence of diaphragm dysfunction appears to be more likely a determinant of early prognosis. Eventually, the co-existence of both diseases was associated with the worse outcome.

## Supplementary information

**Additional file 1: Figure S1.** Kaplan–Meier two-year survival curves in patients with diaphragm dysfunction, intensive care unit-acquired weakness (ICU-AW), no disease or both diseases. **Figure S2.** Kaplan–Meier two-year survival curves in patients with diaphragm dysfunction, intensive care unit-acquired weakness (ICU-AW), no disease or both (sensitivity analysis restricted to hospital survivors). **Figure S3.** Kaplan–Meier two-year survival curves in patients with and without severe diaphragm dysfunction as defined by a change in tracheal pressure in response to magnetic stimulation < 7 cmH2O. **Figure S4.** SF-36 physical component scores at two years among patients with diaphragm dysfunction only, with intensive care unit acquired weakness only, with none of the diseases and with both diseases (panel A) and SF-36 mental component scores among patients with diaphragm dysfunction only, with intensive care unit acquired weakness only, with none of the diseases and with both diseases (panel B) (n = 40 patients).

## Data Availability

The datasets used and/or analysed during the current study are available from the corresponding author on reasonable request.

## Abbreviations

Ptr,stim: tracheal pressure in response to bilateral magnetic stimulation of the phrenic nerves
ICU: intensive care unit
HRQOL: health-related quality of life
SF-36: Short Form (36) Health Survey
