# Supplementary material for: ICU-acquired weakness, diaphragm dysfunction and long-term outcomes of critically ill patients
Source: Ann Intensive Care. 2020 Jan 3;10:1. doi: 10.1186/s13613-019-0618-4 (PMC6942110; doi:10.1186/s13613-019-0618-4)

**Impact of ICU-acquired Weakness and Diaphragm Dysfunction on Long-Term Survival of Critically Ill Patients**

Clément Saccheri, Elise Morawiec, Julie Delemazure, Julien Mayaux, Bruno-Pierre Dubé, Thomas Similowski, Alexandre Demoule, Martin Dres

***Additional file 1***

**Figure S1.** Kaplan-Meier two-year survival curves in patients with diaphragm dysfunction, intensive care unit-acquired weakness (ICU-AW), no disease or both diseases.

**Figure S2.** Kaplan-Meier two-year survival curves in patients with diaphragm dysfunction, intensive care unit-acquired weakness (ICU-AW), no disease or both (sensitivity analysis restricted to hospital survivors).

**Figure S3.** Kaplan-Meier two-year survival curves in patients with and without severe diaphragm dysfunction as defined by a change in tracheal pressure in response to magnetic stimulation < 7 cmH2O.

**Figure S4.** SF-36 physical component scores at two years among patients with diaphragm dysfunction only, with intensive care unit acquired weakness only, with none of the diseases and with both diseases (panel A) and SF-36 mental component scores among patients with diaphragm dysfunction only, with intensive care unit acquired weakness only, with none of the diseases and with both diseases (panel B) (n=40 patients).


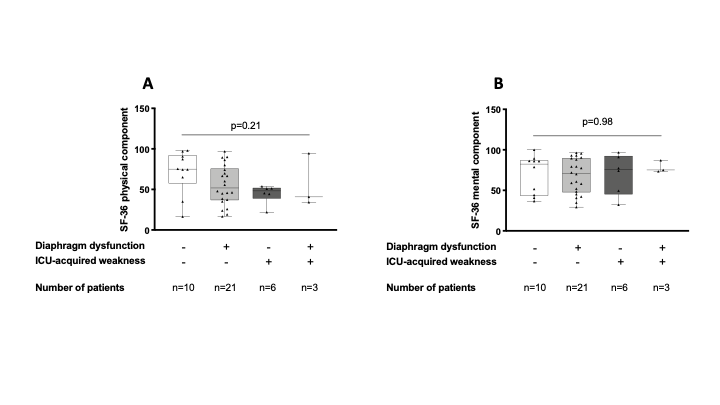

Supplement: Supplementary file 1 — Additional file 1: Figure S1. Kaplan–Meier two-year survival curves in patients with diaphragm dysfunction, intensive care unit-acquired weakness (ICU-AW), no disease or both diseases. Figure S2. Kaplan–Meier two-year survival curves in patients with diaphragm dysfunction, intensive care unit-acquired weakness (ICU-AW), no disease or both (sensitivity analysis restricted to hospital survivors). Figure S3. Kaplan–Meier two-year survival curves in patients with and without severe diaphragm dysfunction as defined by a change in tracheal pressure in response to magnetic stimulation < 7 cmH2O. Figure S4. SF-36 physical component scores at two years among patients with diaphragm dysfunction only, with intensive care unit acquired weakness only, with none of the diseases and with both diseases (panel A) and SF-36 mental component scores among patients with diaphragm dysfunction only, with intensive care unit acquired weakness only, with none of the diseases and with both diseases (panel B) (n = 40 patients). [file 13613_2019_618_MOESM1_ESM.docx]
